# Differential regulation of mRNAs and lncRNAs related to lipid metabolism in Duolang and Small Tail Han sheep

**DOI:** 10.1038/s41598-022-15318-z

**Published:** 2022-07-01

**Authors:** Tianyi Liu, Hui Feng, Salsabeel Yousuf, Lingli Xie, Xiangyang Miao

**Affiliations:** State Key Laboratory of Animal Nutrition, Institute of Animal Sciences, Chinese Academy of Agricultural Sciences, Beijing, 100193 China

**Keywords:** Biochemistry, Computational biology and bioinformatics, Molecular biology, Zoology, Biomarkers, Diseases

## Abstract

The function of long non-coding RNA (lncRNA) can be achieved through the regulation of target genes, and the deposition of fat is regulated by lncRNA. Fat has an important effect on meat quality. However, there are relatively few studies on lncRNAs in the subcutaneous adipose tissue of Duolang sheep and Small Tail Han sheep. In this study, RNA-Seq technology and bioinformatics methods were used to identify and analyze the lncRNA and mRNA in the subcutaneous adipose tissue of the two breeds of sheep. The results showed that 107 lnRNAs and 1329 mRNAs were differentially expressed. The differentially expressed genes and lncRNA target genes were significantly enriched in the biosynthesis of unsaturated fatty acids signaling pathway, fatty acid metabolism, adipocyte differentiation and other processes related to fat deposition. Among them, LOC105616076, LOC114118103, LOC105607837, LOC101116622, and LOC105603235 target FADS1, SCD, ELOVL6, HSD17B12 and HACD2, respectively. They play a key regulatory role in the biosynthesis of unsaturated fatty acids. This study lays a foundation for the study of the molecular mechanism of lncRNA on fat development, and has reference value for studying the differences in fat deposition between Duolang sheep and Small Tail Han sheep.

## Introduction

Sheep can provide us with important resources such as skin, wool and milk, and mutton is an important part of the dietary structure of our residents^1,2^. Different sheep breeds have different fat deposits. This has important effects in the process of sheep breeding, including the effects on meat quality, reproductive performance, fattening efficiency and environmental adaptability. In addition, adipose tissue can also secrete a variety of cytokines to regulate the body’s metabolic balance^3^. Through the study of adipose tissue, we can effectively solve the problem of long-term pursuit of high lean meat percentages resulting in lower meat quality. In addition, this can provide a treatment basis for obesity-induced obesity^4^, hyperlipidemia^5^, fatty liver^6^ and other metabolic diseases, and cardiovascular system diseases^7^. Transcription factors that can regulate fat deposition include C/EBPs, IGF2, PPARγ, ADD1, etc.^8,9^. In recent years, high-throughput sequencing technology has continued to develop, and more and more genes and regulatory factors affecting fat deposition have been discovered. Therefore, it is possible to understand the regulation mechanism of adipose tissue more accurately and comprehensively.

LncRNA is a kind of non-coding RNA with a length greater than 200 nt that exists in various species and was once considered "transcription noise"^10^.With the wide application of high-throughput technology, many lncRNAs involved in regulating fat deposition have been found in fat tissues of livestock and poultry. Jiang et al.^11^ obtained 789 known lncRNAs and 2927 new lncRNAs in the subcutaneous fat tissue of Qinchuan cattle at different stages. Wang et al.^12^ found that lnc-OAD can regulate 3T3-L1 adipocyte differentiation, and regulate fat formation by affecting mitotic clone expansion and regulating WNT/β-catenin signaling pathway. The above studies indicate that many new lncRNAs have been predicted. Those lncRNAs may be involved in the differentiation process of animal fat cells. Huang et al. performed RNA-Seq analysis on the subcutaneous fat tissue of Laiwu pigs and Large White pigs. They identified 54 differentially expressed lncRNAs, whose target genes were significantly enriched in the PPAR signaling pathway^13^. Ma et al.^14^ analyzed the expression profiles of lncRNA and mRNA of different tail types of sheep fat. They found that the differentially expressed lncRNA target genes were enriched in fatty acid metabolism and fatty acid elongation related pathways to promote fat deposition. It shows that lncRNA plays an important regulatory role in fat deposition and fatty acid metabolism in livestock and poultry. Han et al.^15^ analyzed the lncRNA and mRNA expression profiles of intramuscular fat in Aohan fine-wool sheep at different stages, and found the candidate lncRNAs regulate fat deposition by target genes. Cheng et al.^16^ compared differences between preadipocytes and mature adipocytes by whole—transcriptome sequencing and constructed systematically regulatory networks according to the relationship predicted among the differentially expressed RNAs. They found that the lncRNAs play important roles in the regulatory networks and influence adipocyte differentiation. These studies indicate that lncRNAs might determine fat deposition and regulate adipogenic differentation. However, there are few studies on the expression profile and function of lncRNA in the subcutaneous adipose tissue of Duolang sheep and Small Tail Han sheep with differences in fat deposition.

Duolang Sheep^17^ is a sheep breed with excellent meat-fat quality in Xinjiang, China. It has the characteristics of high meat yield and strong fecundity. Its meat is tender and juicy, with uniform fat deposition and no taint. Small Tail Han Sheep^18–20^ is a dominant sheep breed in northern China, with high fecundity, early sexual maturity, stable genetic performance, and strong adaptability.Although the Small Tail Han sheep has many advantages, the meat body size is not obvious and the carcass meat production rate is low^21^. In addition, the tail of the Duolang sheep is large and the tail of Small Tail Han sheep is short and round. The two samples belonged to fat-tailed sheep. These two kinds of sheep provide good research objects for adipogenic differentiation and fat deposition. In this study, RNA-Seq technology and bioinformatics methods were used to sequence the transcriptome of lncRNA and mRNA in the subcutaneous adipose tissue of Duolang sheep and Small Tail Han sheep. In addition, we conducted a comprehensive analysis of them to have a deeper understanding of the role of lncRNA in fat deposition and lipid metabolism. These can lay the foundation for revealing the fat development of two breeds of sheep.

## Materials and methods

### Experimental animals and sample preparation

In this study, three healthy female Duolang sheep and three healthy female Small Tail Han sheep were fed a diet formulated to meet current nutritional requirements. All were 2 years old. All animals can drink and eat freely under natural light. The weights of the species were similar (50 kg), and all were healthy and in good physical condition. We collected subcutaneous adipose tissue samples located at the backfat. To ease the pain,we stunned them with electricity and then slaughtered. The subcutaneous adipose tissue was sampled into a 5 ml tube within 30 min after slaughter and immediately frozen in liquid nitrogen. Then transferred to refrigerator at -80° for long-term preservation and further total RNA extraction.

### Isolation of total RNA and quality control

Total RNAs were extracted from the same amount of subcutaneous adipose tissue using TRIzol^22^ (Invitrogen Life Technologies, Carlsbad, USA) according to the manufacturer’s instructions. In addition, genomic DNA was removed using rDNAIRnase-free (TaKara). RNA quality was verified using 2100 Bioanalyzer (AgilentTechnologies, Santa Clara, CA, USA) and NanoDrop 2000 (Thermo Scientific, USA). Qualified RNA was used for sequencing library construction, and their OD260/280 ≥ 1.8, OD260/230 ≥ 1.0, RIN ≥ 8, the brightness of the 28S was significantly higher than the 18S, and the RNA concentrations of all samples were 200 ng/μL.

### cDNA library construction and RNA sequencing

The qualified total RNAs were used for library construction. RNA-seq transcriptome strand library was prepared following TruSeq stranded total RNA Kit from Illumina (San Diego, CA) using total RNA. The steps included removal of ribosomal (rRNA) and enrichment of mRNA. Then SuperScript double-stranded cDNA synthesis kit (Invitrogen, CA) was used and first-stranded cDNA was synthesized with random hexamer primers. We removed the RNA template and synthesized a replacement strand, incorporating dUTP in place of dTTP to generate ds cDNA. This was followed by the addition of the end repair mix to blunt the sticky end, followed by the addition of an A base at the 3'end to form a Y-linker. The products after the adaptor were purified and sorted. The sorted products were used for PCR amplification and purification to obtain the final library. High throughput sequencing was conducted using the Illumina NovaSeq 6000 sequencing platform.

### Reference genome mapping and transcriptome assembly

Clean data were obtained using fastp software (V0.19.5) filtering out the raw data containing joints, low-quality reads, N rate (N represents uncertain base information), higher sequences and too short sequences^23^, the remaining sequences were used for further analysis. Comparing clean reads with the reference genome GCF_002742125.1 using Hisat2^24^ (V2.1.0). Analyzed valid data mapped reads. Then, we spliced mapped reads using StringTie^25^ software (V1.3.3b). Comparing with the original genome annotation information, we found unannotated transcription regions and discovered new transcripts and new genes of the species.

### Identification of potential lncRNA candidates

LncRNA is a non-coding RNA with a length of more than 200 nucleotides. Based on these features, we selected the transcripts with class symbols "x", "i", "j", "u", "o". On this basis, the lncRNAs with length ≥ 200 nt, number of exon ≥ 2 and ORF ≤ 300 bp were selected as candidates for preliminary screening. The absence of protein-coding ability was a key condition for judging lncRNAs. We used CPC^26^, CNCI^27^ and Pfam^28^ to eliminate transcripts with coding ability. Therefore, we obtained the newly predicted lncRNA in the fat sample, and identified known lncRNAs from NCBI and Ensembl databases.

### Screening of differentially expressed genes and lncRNAs

The expression level of lncRNAs and mRNAs were corrected by the transcripts per million reads (TPM) method, and the gene length and sequencing depth were uniformized. Therefore, that the expression levels in different samples were consistent. The expression levels between genes can be more intuitively. For experiments with biological replicates, use the DESeq2 based on negative binomial distribution to perform statistical analysis on raw counts, and obtain differential expression based on *p* value < 0.05, |log2FoldChange|> 1.

### GO and KEGG enrichment analyse of differentially expressed genes

Gene Ontology Consortium (GO) can be used for functional enrichment analysis of differentially expressed genes, divided into cellular component (CC), molecular function (MF) and biological process (BP)^29,30^. Kyoto Encyclopedia of Genes and Genomes (KEGG)^31^ can link genomic and functional information. Classification of functions was exercised^32^. We displayed the genes in the gene set on the KEGG pathway diagram, and display the KEGG annotation pathway diagram they participate in. The multiple test correction method Benjamini and Hochberg (BH) was used to correct the pvalue, resulting in padjust. When the *p*adjust < 0.05, it was significantly enriched.

### Protein–protein interaction (PPI) network analysis for differentially expressed genes

According to the STRING database (http://stringdb.org/), the PPI network analysis of differentially expressed genes was carried out. The relationship between the differentially expressed genes in the subcutaneous adipose tissue of Duolang sheep and Small Tail Han sheep was further studied. The visually analyze was conducted using Cytoscape software^33^ with the obtained differential gene encoding protein interaction network data files, thereby obtaining key genes.

### Prediction and functional analysis of the target genes of differentially expressed lncRNAs

LncRNA is a non-coding RNA whose function is mainly to regulate target genes. According to the different mode of action of lncRNA, it is divided into homeopathic regulation (cis-regulate) and trans-regulation (trans-regulate). Homeopathic regulation and trans regulation of distant protein-coding genes were mentioned in Wang et al.^34^. The co-expression relationship between lncRNA and mRNA was realized by Pearson correlation coefficient (PCC) calculation. The setting standard was |PCC|> 0.8 and *p* value < 0.05 to screened the co-expressed lncRNA-mRNA. When screening target genes, genes within 100 kb of lncRNA were considered as target genes for cis^35^. Genes with |PCC|> 0.9 and a significant pvalue less than 0.05 in the co-expression analysis were used as target genes for the trans-regulate of differential lncRNAs. The potential role of lncRNA was studied through GO annotation and KEGG pathway enrichment analysis of target genes.

### Real-time fluorescence-based quantitative PCR (qRT-PCR) verification

QRT-PCR was used to verify the reliability of the sequencing results. Seven differentially expressed mRNAs and six differentially expressed lncRNAs were randomly selected. These genes come from PPI, lncRNA-mRNA network and fat metabolism related pathways. Three biological replicates were employed for each gene. 0.5 μg RNA was taken to synthesize cDNA template through GeneAmp PCR System 9700 (APPlied Biosystems, USA). The qRT-PCR analysis was conducted using LightCycler 480IIReal-time PCR Instrument (Roche, Swiss). The reaction system consisted of 1 μL cDNA, 5 μL of 2 × PerfectStart Green qPCR SuperMix, 0.2 μL of 10 μM forward primer, 0.2 μL of 10 μM reverse primer, and 3.6 μL of nuclease-free H2O. Reaction conditions were as follows: 94 °C for 30 s, 94 °C for 5 s, 60 °C for 30 s, 45 cycles. After the cycle, the melting curve was used to detect the specificity of the product: the temperature was slowly increased from 60 to 97 °C. The relative expression levels of genes between samples were calculated using 2−ΔΔCt method^36^. Data obtained were analyzed using GraphPad Prism (V8.0.1). The student t-test (*p* < 0.05) was used for mean comparisons. All results were presented in bar charts with the means and their standard deviation (± SD). Primers used for the qRT-PCR are listed in supplementary table 6.

### Statistical analysis

All the data were presented as means ± SD. When comparisons were made, a Student’s t-test was performed and *p* value < 0.05 was considered as statistically significant.

### Ethics statement

All the procedures involving animals were approved by the animal care and use committee at the Institute of Animal Sciences, Chinese Academy of Agricultural Sciences (NO. IAS2019-82), where the study was conducted. All the experiments were performed in accordance with the relevant guidelines and regulations set by the Ministry of Agriculture of the People’s Republic of China. This study was carried out in compliance with the ARRIVE guidelines.

## Result

### Total RNA sequencing mapping

To comprehensively understand the transcriptomes of subcutaneous fat tissue in Duolang sheep and Small Tail Han sheep, total RNAs were isolated and sequenced through Illumina sequencing platform. Approximately 124 Gb raw data were obtained for each sample. Specifically, 136,631,686, 141,871,442, and 144,353,224 raw reads were obtained for Duolang sheep (D-PF-1, 2, and 3, respectively); 137,953,526, 129,535,766, 132,333,836 raw reads were obtained for Small Tail Han sheep (X-PF-1, 2, and 3, respectively) (Table 1). The raw reads were filtered to obtain clean reads, which were mapped to the Ovis aries GCF_002742125.1 of the sheep genome sequence, with the mapping ratio ranging from 94.69 to 96.46%. The transcripts were assembled using String Tie (V1.3.3b) with default parameter. The results of the RNA-Seq reads mapped on the reference are shown in Table 1.

**Table 1:** Summary of raw reads after quality control and mapping to the reference genome.

Sample	Raw Reads	Clean Reads	Clean ratio (%)	Q30 (%)	GC content (%)	Total mapped	Unique mappedc
D-PF-1	136,631,686	135,393,598	99.09	95.22	52.05	130,223,053 (96.18%)	99,220,652 (73.28%)
D-PF-2	141,871,442	140,587,412	99.09	95.01	52.78	133,116,845 (94.69%)	106,911,296 (76.05%)
D-PF-3	144,353,224	142,895,188	98.99	95.23	52.09	137,830,324 (96.46%)	103,526,375 (72.45%)
X-PF-1	137,953,526	136,746,686	99.13	95.38	52.25	131,380,772 (96.08%)	101,534,519 (74.25%)
X-PF-2	129,535,766	128,433,640	99.15	95.2	52.45	123,830,160 (96.42%)	95,751,126 (74.55%)
X-PF-3	132,333,836	130,999,204	98.99	95.17	52.23	125,839,893 (96.06%)	97,392,935 (74.35%)

### Identification and characterization of lncRNAs in subcutaneous adipose tissue of sheep

**Figure 1:**
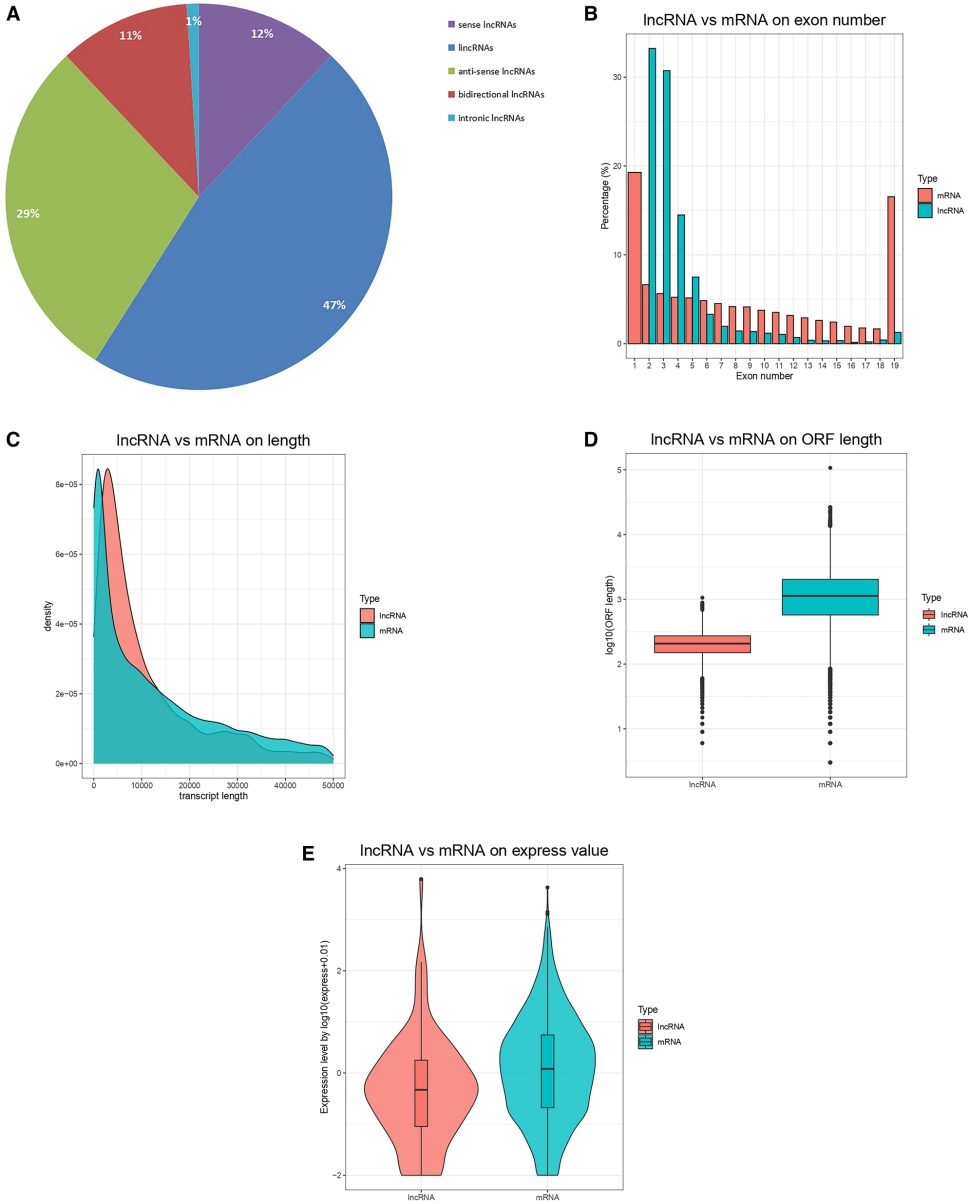
LncRNA characterization and gene expression. (**A**) Summary of lncRNA types. (**B**) Number distribution of lncRNA and mRNA exons. (**C**) LncRNA and mRNA length distribution. (**D**) LncRNA and mRNA ORF length. (**E**) Expression levels of lncRNA and mRNA.

According to the positional relationship between lncRNAs and protein coding genes, they are classified into five categories^37,38^. A total of 4464 lncRNAs candidates in the six libraries were identified, including 2094 lincRNAs, 1290 anti-sense lncRNAs, 534 sense lncRNAs, 491 bidirectional lncRNAs and 55 intronic lncRNAs (Fig. 1A). Most lncRNAs have 2 to 4 exons, while mRNAs have 6 or more exons than lncRNAs. Most lncRNAs have 19 exons (Fig. 1B). In Fig. 1C, the comparison shows that the lengths of lncRNA and mRNA have the same increasing and decreasing trend. However, the ratio of longer lncRNA is lower than that of mRNA. It is predicted that the open reading frame length of lncRNA is generally shorter than that of mRNA, as shown in Fig. 1D.

### Expression level of genes and differential expression analysis

According to the transcript expression level measurement index TPM value, a violin plot was constructed, and the transcript expression levels in the two samples were analyzed separately (Fig. 1E). A total of 4464 lncRNAs and 40,278 mRNAs, including the newly predicted protein coding genes were found. The concentration of mRNA expression was greater than that of lncRNA, indicating that the expression level of lncRNA was lower. Among them, there were 107 lncRNAs (Supplementary Table 1), and 1329 mRNAs were differentially expressed in the subcutaneous adipose tissue of Duolang sheep and Small Tail Han sheep (Supplementary Table 2).

### GO analysis of differentially expressed genes

**Figure 2:**
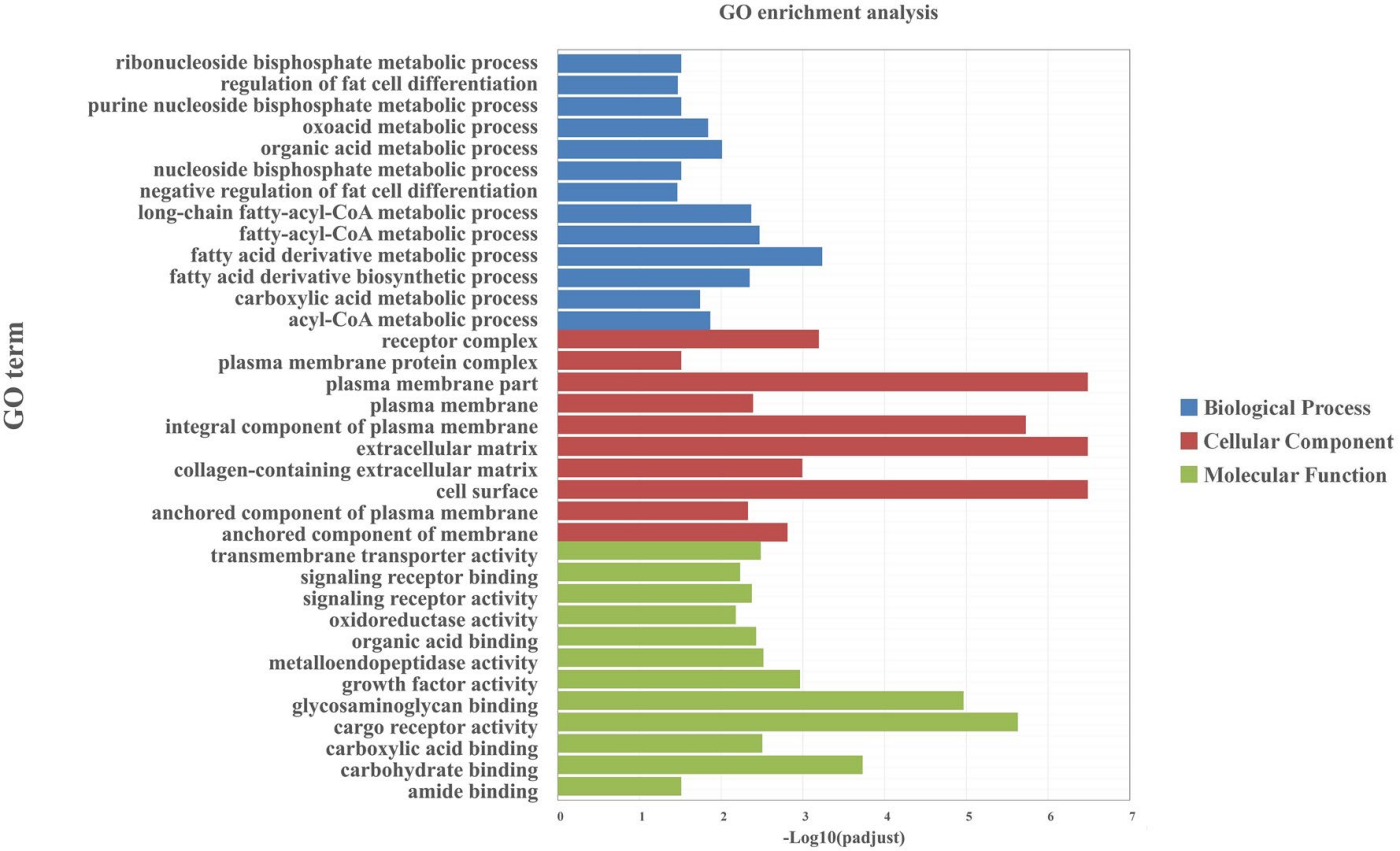
GO analysis of differentially expressed genes. The figure is composed of three parts: biological processes, molecular functions, and cellular components.

In order to understand the function of differentially expressed genes and gene products, GO annotation was used to analyze the biological process (BP), molecular function (MF) and cell composition (CC) of differentially expressed genes. Among the 1329 differentially expressed genes in the subcutaneous adipose tissue of Duolang sheep and Small Tail Han sheep, there are 1135 genes annotated with GO term (Supplementary Table 2). In Fig. 2, the regulation of multicellular organismal process and the positive regulation of multicellular organismal process are the two most important enrichment processes in biological processes, followed by fatty acid derivative metabolic process, regulation of fat cell differentiation and significantly enrichment of fatty-acyl-CoA in metabolic process. In molecular functions, it is mainly enriched in items such as cargo receptor activity and carbohydrate binding. In cell components, it is mainly enriched in items such as membrane part and intrinsic component of membrane. Through GO enrichment analysis, it is found that the differentially expressed genes mainly regulate the signal transduction and lipid metabolism processes of the adipocytes of Duolang sheep and Small Tail Han sheep.

### KEGG enrichment analysis of differentially expressed genes

**Figure 3:**
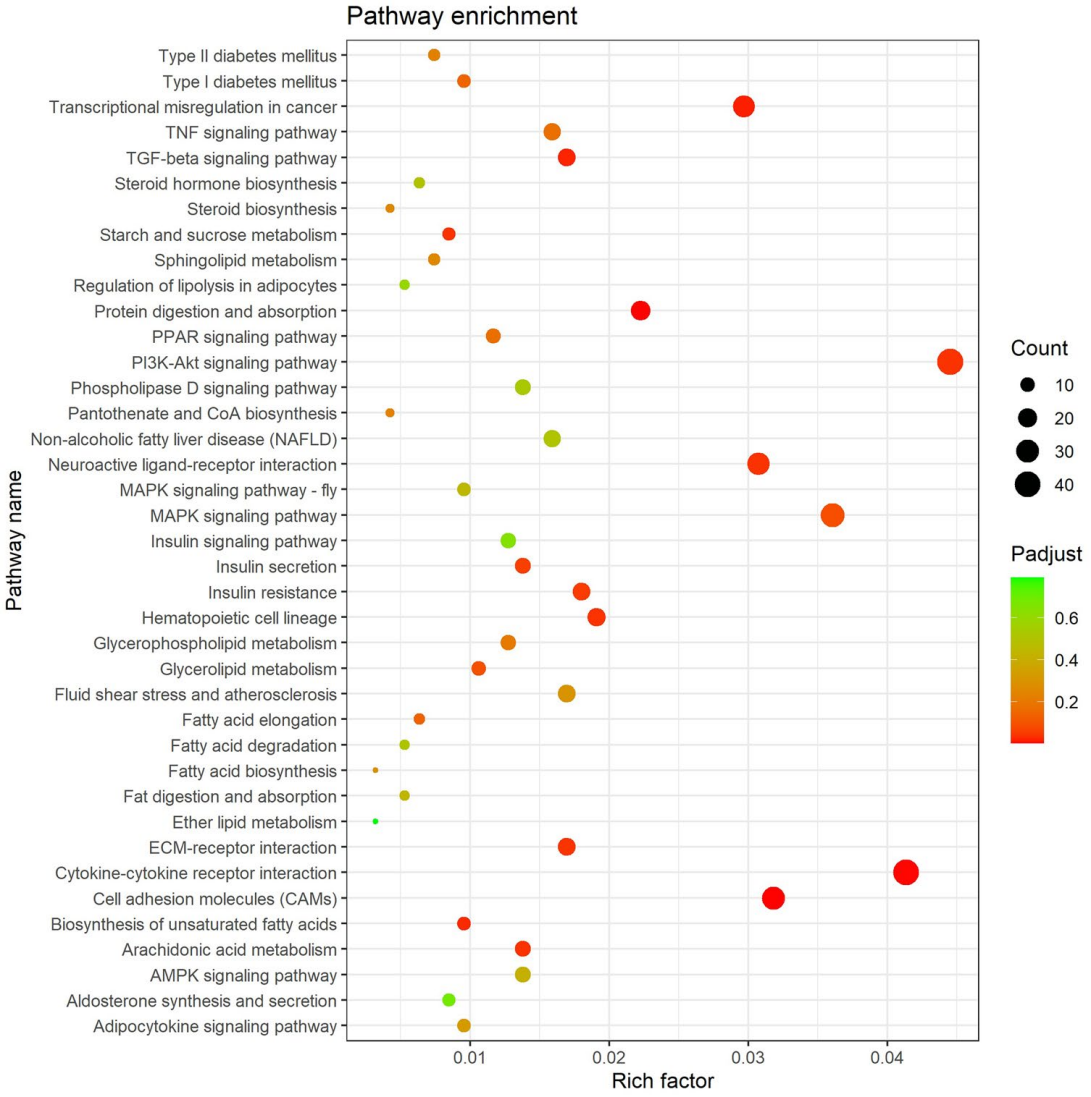
KEGG pathway analysis of differentially expressed genes. Y-axis label represents pathway, and X-axis label represents rich factor (rich factor = amount of differentially expressed genes enriched in the pathway/amount of all genes in background gene set). The size and color of the bubble represent the number of differentially expressed genes enriched in the pathway and significance of enrichment, respectively.

The results of KEGG enrichment analysis showed that a total of 943 out of 1329 differentially expressed genes were annotated with KEGG. They were annotated into 306 signaling pathways, of which 20 pathways were significantly enriched (Supplementary Table 2). In Fig. 3, pathway enrichment results show that differentially expressed genes are enriched in a large number of pathways related to lipid metabolism, including biosynthesis of unsaturated fatty acids, arachidonic acid metabolism, glycerolipid metabolism, etc. In addition, differentially expressed genes are also significantly enriched in cell signal transduction pathways such as PI3K-Akt and TGF-beta signaling pathway to regulate proliferation, differentiation and apoptosis. In addition, the differentially expressed genes were also significantly enriched in insulin resistance related diseases caused by abnormal lipid metabolism. The above results indicate that the differentially expressed genes in the subcutaneous adipose tissue of Duolang sheep and Small Tail Han sheep are involved in multiple pathways related to lipid metabolism. Among them, genes enriched in biosynthesis of unsaturated fatty acids and arachidonic acid metabolism may be beneficial to Duolang sheep. It plays an important role in regulating the metabolism of subcutaneous fat in Small Tail Han sheep.

### PPI network for differentially expressed genes

**Figure 4:**
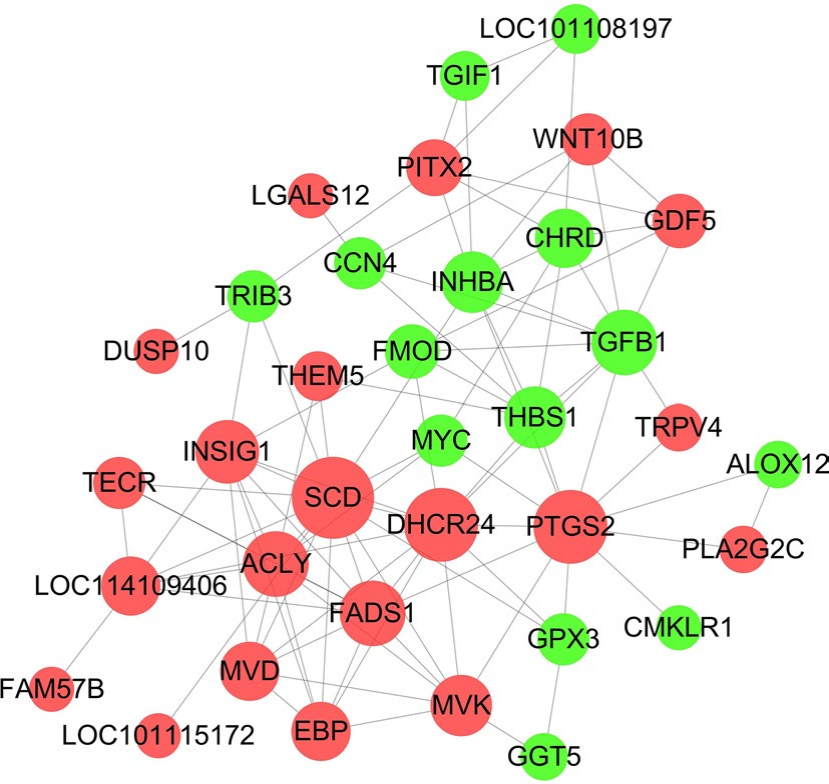
PPI network of differentially expressed genes related to lipid metabolism. Node represents protein, edge represents interaction between proteins. The size of the node is proportional to the degree of the node (the degree of the node is defined as the number of proteins interacting with this node). Red represents the up-regulation of Duolang sheep, and green represents the down-regulation.

Fat deposition is the result of fat cell differentiation and lipid metabolism. In this study, a PPI network of differentially expressed genes related to adipocyte differentiation and lipid metabolism was constructed through PPI network analysis and gene differential expression. In addition, genes that regulate Duolang sheep fat deposition and Small Tail Han sheep were determined (Supplementary Table 3). In the PPI network, combine-score ≥ 0.6 and degree ≥ 10 were used as thresholds to screen key nodes. Among them, SCD, DHCR24, PTGS2, TGFB1, FADS1 and other encoded proteins were at the key node positions (Fig. 4), which may be involved in subcutaneous fat metabolism. This has an important regulatory role in adipogenic differentiation.

### Target genes of lncRNAs and functional analysis

**Figure 5:**
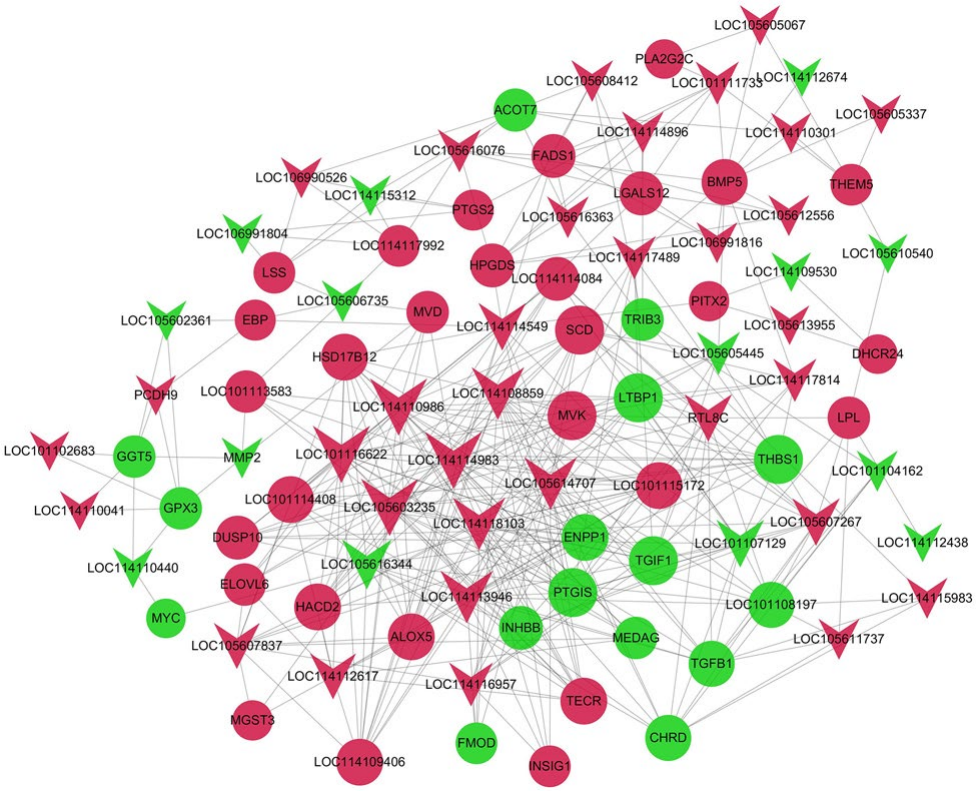
Target relationship between lncRNA and mRNA. The circle in the node represents mRNA, and the triangle represents lncRNA, edge represents interaction between lncRNA and mRNA. In addition, red represents up-regulation of Duolang sheep, green represents down-regulation.

The differentially expressed lncRNAs in the subcutaneous adipose tissue of Duolang sheep and Small Tail Han sheep were used to predict the target genes of cis and trans. The results showed that 18 differentially expressed lncRNAs were predicted to target 28 genes in line with cis action, of which 10 were differentially expressed target genes (Table 2). The prediction results of trans target genes showed that 56 of the 107 differentially expressed lncRNAs are co-expressed with differential mRNAs (Supplementary Table 4). Combining GO and KEGG analysis to screen out the genes related to adipocyte differentiation, lipid metabolism and related diseases, and screen with |PCC|≥ 0.9 as the threshold to construct a co-expression network for the trans-target genes of differential lncRNAs..We found that lncRNA may correspond to multiple mRNAs, and one mRNA may correspond to multiple lncRNAs, and the relationship between the two is not necessarily one-to-one. In Fig. 5, LOC101116622, LOC105603235, LOC114110986, LOC114114983, LOC114118103, LOC114108859, LOC114113946, LOC105614707, LOC105616344 are key lncRNAs.

**Table 2:** Prediction of differentially expressed lncRNA target genes.

lncRNA id	LncRNA regulation	Target gene id	mRNA regulation
MSTRG.15701.2	Down	Gene-TMC6	Up
MSTRG.19616.1	Down	Gene-MMP2	Down
MSTRG.28964.2	Up	Gene-LOC101111733	Up
MSTRG.28964.3	Up	Gene-LOC101111733	Up
rna-XR_001024168.2	Up	Gene-FLYWCH2	Up
rna-XR_001436365.2	Down	Gene-ARMC12	Down
rna-XR_001436365.2	Down	Gene-FKBP5	Down
rna-XR_003585604.1	Up	Gene-SLC25A1	Up
rna-XR_003589506.1	Up	Gene-TMEM59L	Down
rna-XR_003589506.1	Up	Gene-CRLF1	Down
rna-XR_003590087.1	Up	Gene-DPYD	Up

**Figure 6:**
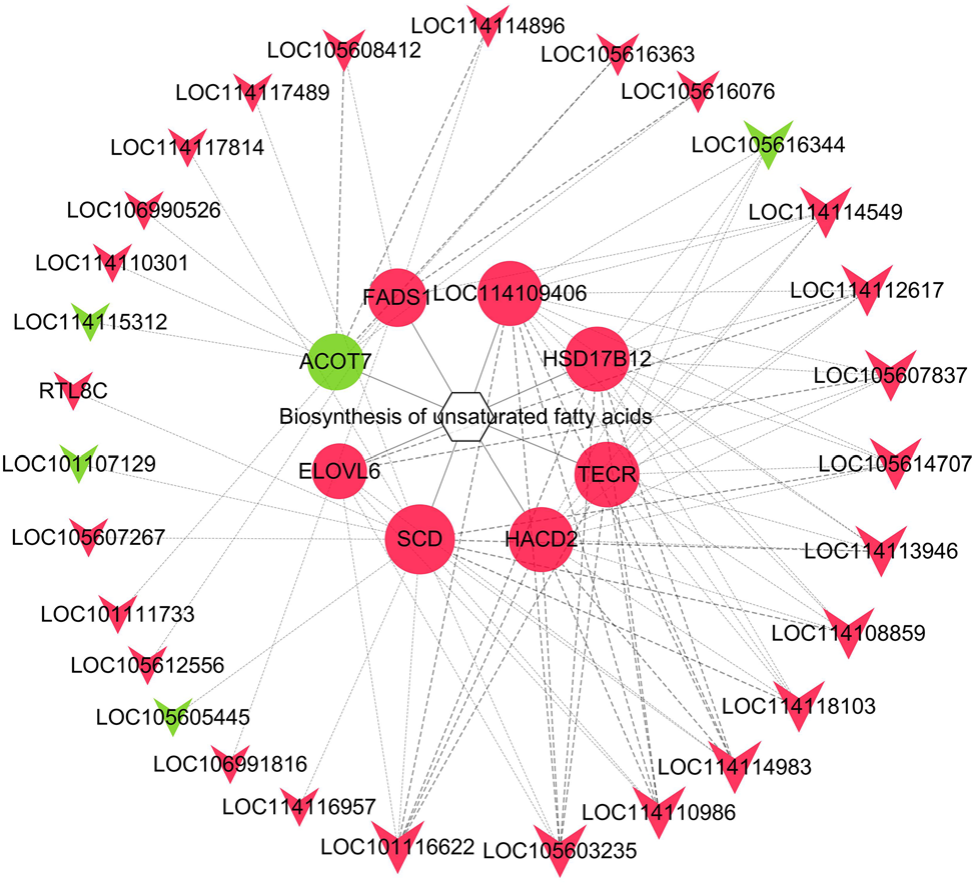
lncRNA biosynthesis of unsaturated fatty acids network. Inverted triangles, circles and hexagons represent lncRNAs, genes and pathways, respectively. Red represents up-regulation, and green represents down-regulation. The edge represents the interaction strength.

The function of the lncRNA can be inferred from the function of mRNA. The GO annotation and KEGG pathway enrichment of the target gene showed that it is significantly enriched in biological processes, including regulation of fat cell differentiation, lipid metabolism, fatty acid metabolism, and arachidonic acid metabolism, lipid metabolism pathway such as biosynthesis of unsaturated fatty acids. This indicates that the differentially expressed lncRNAs may regulate adipose tissue by participating in the above-mentioned signal pathways and biological processes. The target genes were selected to construct a lncRNA-mRNA network in biosynthesis of unsaturated fatty acids (Fig. 6) (Supplementary Table 5), and to explore the lncRNA-mRNA regulation mode.

### qRT-PCR verification

**Figure 7:**
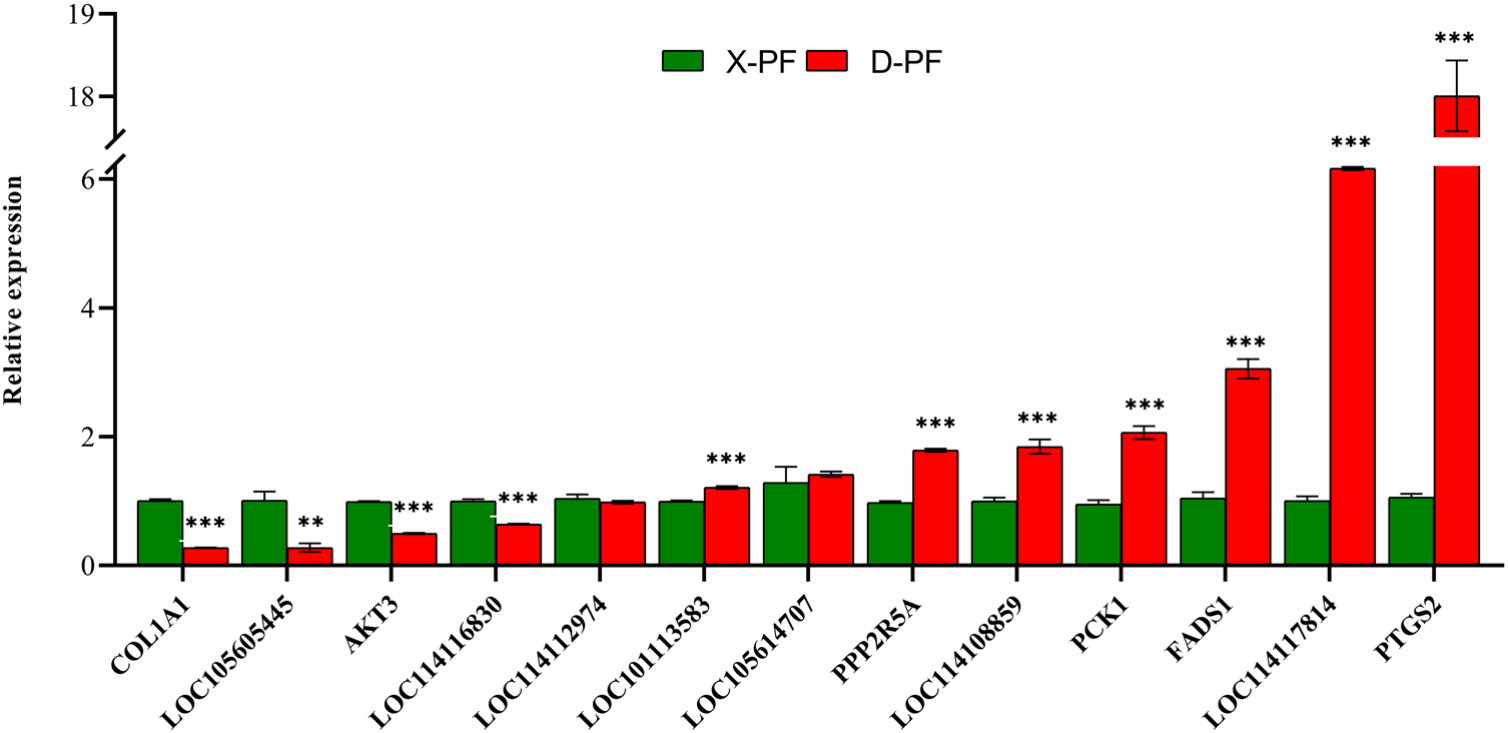
qRT-PCR verification of differentially expressed genes. The differential expression of genes in subcutaneous adipose tissue between Duolang and Small Tail Han sheep was verified qRT-PCR. D-PF represents the subcutaneous adipose tissue of Duolang sheep. X-PF represents the subcutaneous adipose tissue of Small Tail Han sheep. *P value < 0.05; **P value < 0.01; ***P value < 0.001.

QRT-PCR is considered as the golden standard for quantitative analysis of genes. RNA-Seq data is significantly correlated with qRT-PCR results. Therefore, 13 differentially expressed genes were randomly selected to verify the RNA-Seq results. In Fig. 7, LOC101113583, LOC105614707, PPP2R5A, LOC114108859, PCK1, FADS1, LOC114117814 and PTGS2 were up-regulated in subcutaneous adipose tissue of Duolang sheep. COL1A1, LOC105605445, AKT3, LOC114116830 and LOC114112974 were up-regulated in subcutaneous adipose tissue of Duolang sheep. These results were consistent with the sequencing results, indicating the reliability of sequencing results.

## Discussion

Fat deposition is regulated by many transcription factors, key genes and signaling pathways. This study applied RNA-Seq technology to analyze gene expression and reveal its biological characteristics. Duolang sheep and Small Tail Han sheep are high-quality local sheep breeds in China. In order to explore their differences in fat deposition, we use RNA-Seq technology to sequence the subcutaneous fat tissue. Then, we screened out the differentially expressed genes, and performed GO annotation and KEGG enrichment analysis on the 1329 differentially expressed genes. Finally, we screened out the target genes for fat deposit effects through analysis. In addition, the 107 differentially expressed lncRNAs and differentially expressed mRNAs were screened for co-expression analysis. Genes related to adipocyte differentiation, lipid metabolism and related diseases were screened out as the target genes of lncRNAs to reveal the function of lncRNAs. These genes will guide the breeding of high-quality livestock and poultry, improve meat quality, and provide potential targets for the treatment of fat metabolism-related diseases.

The fat composition of the diet plays an important role in maintaining human health and preventing lipid-related diseases. Fatty acids are an important part of lipids, especially unsaturated fatty acids are considered to have important nutritional value^39,40^. In this study, unsaturated fatty acids biosynthesis was significantly enriched with 8 differentially expressed genes, including TECR, ACOT7, LOC114109406, FADS1, SCD, ELOVL6, HSD17B12, HACD2. These genes were regulated by 29 lncRNAs. TECR is a gene related to fatty acid synthesis, which would decrease the transcript level in NaF solution^41^. ACOT7 is an enzyme that catalyze the hydrolysis of acyl-CoAs to free fatty acids and CoA^42^. However, there were no report on TECR, ACOT7 and LOC114109406 gene regulating fat deposition in sheep. FADS1 and SCD are the key node position of PPI network. FADS1 is a member of the fatty acid dehydrogenase gene family and is related to all lipids^43^. It can encode Delta-5 (D5D) desaturase, which is the rate-limiting enzyme for the conversion of polyunsaturated fatty acids. This is considered as the main determinant of the level of polyunsaturated fatty acids^44^. That the expression of FADS1 in the lower adipose tissue of the cowhide is up-regulated under cold stimulation conditions. This promotes the production of polyunsaturated fatty acids in the lower adipose of the cowhide^45^. It was also confirmed in pigs and cattle^46,47^. In addition, FADS1 is also associated with metabolic diseases (obesity, metabolic syndrome) and cardiovascular diseases (arterial hypertension, coronary heart disease)^48,49^, In this study, FADS1 was up-regulated in Duolang sheep, which was consistent with previous study showing that FADS1 was highly expressed in subcutaneous adipose tissue of other species. Meanwhile, LOC105616076 might target FADS1 and involve in unsaturated fatty acids biosynthesis, thus regulating lipid metabolism.

Stearoyl-CoA desaturase (SCD) is an endoplasmic reticulum binding enzyme, belonging to the dehydrogenase family. It is the key enzyme that catalyzes the formation of unsaturated fatty acids from saturated fatty acids, especially palmitoleic acid and oleic acid. The high expression of SCD can promote the fat production, and the expression product can participate in the differentiation of pre-adipocytes and cell metabolism. There are many subtypes of SCD, among which SCD1 is a downstream gene regulated by SREBP1. When the sterol regulatory element on the SCD1 promoter is combined with the SREBP1c transcription factor, transcription is upregulated and lipid synthesis increases^50^. The backfat thickness of Duolang was higher than that of Small Tail Han sheep. In this study, SCD is up-regulated in the subcutaneous fat tissue of Duolang sheep, which is consistent with previous studies in sheep^51^, cattle^52^ and pig^53^. These results suggest that SCD promotes subcutaneous fat deposition in Duolang sheep and the mechanisms of SCD regulating fat deposition in cattle and pig might be same. In addition, LOC114118103 might participate in the biosynthesis of unsaturated fatty acids pathway by regulating SCD, and regulate the deposition of subcutaneous adipose tissue in Duolang sheep and Small Tail Han sheep.

Long-chain fatty acid elongase 6 (ELOVL6) is one of the important regulatory factors that regulate fatty acid synthesis. It is the rate-limiting enzyme for the elongation of long-chain fatty acids. It mainly regulates cell metabolism, proliferation or apoptosis by affecting the composition of fatty acids and metabolites. The ELOVL6 polymorphism is significantly related to subcutaneous fat deposition in chickens^54^. The expression of the ELOVL6 requires activation of the retinoid X receptor RXR^55^. Its product stearic acid regulates mitochondrial function through transferrin receptor 1 (TFR1)^56^. Lin et al.^57^ analyzed indicated that SCD and ELOVL6 expressions were positively correlated with the concentrations of polyunsaturated fatty acids in yak subcutaneous fat. However, there is no report on ELOVL6 gene regulating fat deposition in sheep. In this study, ELOVL6 was used as the target gene of LOC105607837 to regulate fat deposition. HSD17B12 is a multifunctional isoenzyme, which plays an important role in the elongation of long-chain fatty acids. Increased gene transcription of HSD17B12 may lead to increased lipid synthesis. This can reduce 3-ketoacyl-CoA in the prolongation of VLCFA biosynthesis. It can be catalytically reduced to 3-hydroxyacyl-CoA^58,59^. In this paper, LOC101116622 regulated lipid metabolism by targeting HSD17B12. HACD2 is the main 3-hydroxyacyl-CoA dehydratase. It produces HACD2-deficient cells in the mammalian system, resulting in the reduction of ≥ C18 saturated and monounsaturated FAs^60^. This proves that HACD2 contributes to fat deposition. In this paper, LOC105603235 regulated the metabolism of fatty acids by targeting the HACD2, resulting in thicker backfat in Duolang sheep.

The above studies, have shown that through the identification and analysis of genes in the subcutaneous adipose tissue of Duolang sheep and Small Tail Han sheep, a number of candidate genes involved in the biosynthesis of unsaturated fatty acids have been screened, including TECR, ACOT7, LOC114109406, FADS1, SCD, ELOVL6, HSD17B12 and HACD2. These genes may have reference value for studying the difference in fat deposition between Duolang sheep and Small Tail Han sheep.

## Conclusions

In this study, RNA-Seq technology and bioinformatics methods were applied to explore the mechanism of fat deposition in sheep. We identified the differentially expressed lncRNAs and genes of subcutaneous adipose tissue between Duolang and Small Tail Han sheep. On this basis, 107 lncRNAs and 1329 mRNAs were differentially expressed. Combined with GO and KEGG analysis of differentially expressed genes, lncRNA participates in the biosynthesis of unsaturated fatty acids pathway through targeted mRNA. By this way, they regulated subcutaneous fat deposition in Duolang and Small Tail Han sheep. LOC105616076, LOC114118103, LOC105607837, LOC101116622 and LOC105603235, which might target FADS1, SCD, ELOVL6, HSD17B12 and HACD2, respectively, play key regulatory roles. Previous studies on lncRNAs have mostly appeared in model animals such as mice, or in pigs that are genetically similar to humans, and there is relatively few researches on lncRNAs in sheep. This study can provide useful information for understanding molecular mechanism of fat deposition in sheep. In addition, this study can help breed sheep with high meat quality as well as prevent and treat disease associated with fat metabolism.

## Supplementary Information

Supplementary Information 1.

Supplementary Information 2.

Supplementary Information 3.

Supplementary Information 4.

Supplementary Information 5.

Supplementary Information 6.

Supplementary Tables Legends.

## Data Availability

The RNA-Seq data were submitted to SRA database under accession number (SRR15371345, SRR15371346, SRR15371347, SRR15371348, SRR15371349, SRR15371350, SRR15371351, SRR15371352, SRR15371353, SRR15371354, SRR15371355, SRR15371356). RNA-Seq data have been deposition in the NCBI Sequence Read Archive (SRA) database with accession number PRJNA752760.
