# Supplementary material for: Differential regulation of mRNAs and lncRNAs related to lipid metabolism in Duolang and Small Tail Han sheep
Source: Sci Rep. 2022 Jul 1;12:11157. doi: 10.1038/s41598-022-15318-z (PMC9249921; doi:10.1038/s41598-022-15318-z)
Supplement: Supplementary file 7 — Supplementary Tables Legends. [file 41598_2022_15318_MOESM7_ESM.docx]

Supplementary Tables Legends:

Supplementary Table 1S | lncRNAs were differentially expressed in the subcutaneous adipose tissue

Supplementary Table 2S | Differentially expressed mRNAs and functional analysis

Supplementary Table 3S | Candidate genes for fat deposition

Supplementary Table 4S | Gene coexpression

Supplementary Table 5S | lncRNA-mRNA network in Biosynthesis of unsaturated fatty acids

Supplementary Table 6S | qRT-PCR
